# The diagnostic accuracy of a laser fluorescence device and digital radiography in detecting approximal caries lesions in posterior permanent teeth: an in vivo study

**DOI:** 10.1007/s10103-017-2157-2

**Published:** 2017-02-14

**Authors:** R. Menem, I. Barngkgei, N. Beiruti, I. Al Haffar, Easter Joury

**Affiliations:** 1 Oral Medicine Department, Faculty of Dentistry, Damascus University, Mazzeh highway, Damascus, Syria; 2 School Health Department, Jisser Alabiad Square, P.O Box 60184, Damascus, Syria; 3 Population and Patient Health, King’s College London Dental Institute, Denmark Hill Campus, Bessemer Road, London, SE5 9RS UK

**Keywords:** Caries detection, Approximal surfaces, Laser fluorescence, DIAGNOdent pen, Digital bitewing radiography, Diagnostic accuracy

## Abstract

The aim of this in vivo study was to test the diagnostic accuracy of a pen-type laser fluorescence (LFpen) device in detecting approximal caries lesions, in posterior permanent teeth, at the cavitation and non-cavitation thresholds, and compare it with that of digital bitewing radiography. Thirty patients (aged 18–37), who attended the Faculty of Dentistry at Damascus University for a dental examination, were consecutively screened. Ninety approximal surfaces of posterior permanent teeth without frank cavitations, enamel hypoplasia or restorations were selected and examined using the LFpen (DIAGNOdent pen) and digital bitewing radiography. The reference standard was the visual-tactile inspection, after performing temporary tooth separation, using orthodontic rubber rings, placed for 7 days. The status of included approximal surfaces was recorded as intact/sound, with white/brown spots or cavitated. One trained examiner performed all examinations. There were statistically significant differences in LFpen readings between the three types of approximal surface status (*P* < 0.001). The optimal cut-off values for detecting approximal caries lesions in posterior permanent teeth were >16 and 8 at the cavitation and non-cavitation thresholds respectively. The sensitivity, specificity and accuracy (measured by the area under the receiver-operating characteristic curve) were 100, 85 and 95 and 92, 90 and 95% at the cavitation and non-cavitation thresholds respectively. The intra-class correlation coefficient for intra-examiner reliability was 0.95. The diagnostic accuracy of the LFpen was significantly higher than that of digital bitewing radiography (*P* < 0.001). The LFpen’s diagnostic performance was accurate and significantly better than digital bitewing radiography in detecting approximal caries lesions, in posterior permanent teeth.

**Electronic supplementary material:**

The online version of this article (doi:10.1007/s10103-017-2157-2) contains supplementary material, which is available to authorized users.

## Introduction

The ability to detect caries and discriminate between cavitated and non-cavitated caries lesions is profoundly important in the modern principles of caries lesion management [1]. This ability aids the dentist in decision-making regarding the type of intervention needed [2].

For approximal surfaces of the posterior teeth, the detection and discrimination between cavitated and non-cavitated caries lesions, using the direct visual-tactile inspection, are often hampered by the presence of adjacent teeth. Therefore, temporary tooth separation for a period of approximately 1 week can allow for a direct visual-tactile inspection of approximal surfaces. However, the main disadvantage of this technique is that it needs two appointments to make a diagnosis and it might provoke a greater level of patient’s pain and discomfort compared with other methods [3]. The highest level of pain intensity was reported in the first 2 days after the insertion of separators.

Up to date, bitewing radiography has been the standard aid method for the detection of approximal caries [4, 5]. This method has many limitations. It cannot distinguish the cavitation status of the caries lesion [6]. Its sensitivity is low, particularly at early stages. In addition, it involves small but detectable hazards of exposing individuals frequently to ionising radiation [7]. A systematic review of the literature has synthesised the results of 18 in vivo studies, which investigated the correlation between bitewing radiolucency and cavitation status in approximal caries in permanent teeth [8]. No article presented strong evidence for concluding a clinical threshold at which the restoration of approximal caries is recommended. Overall, the evidence suggested that once radiolucency presents in the dentine, the probability of cavitation increases significantly making it a potential threshold for restoration. It is worth mentioning that none of the reviewed studies has carried out an accuracy test (receiver operating characteristic analysis) to support the validity of such proposed threshold.

Many non-ionising techniques, such as, fibre-optic transillumination (FOTI), quantitative light-induced fluorescence (QLF) and electrical conductance (EC) have been tested for their diagnostic accuracy in detecting approximal caries [9–11].

A new laser fluorescence device (DIAGNOdent pen, KaVo, Biberach, Germany) was developed recently with a tip for the detection of approximal caries [5]. Appendix 1 summarises the previous four in vivo studies that aimed to investigate the validity of this pen-type laser fluorescence (LFpen) device in distinguishing between cavitated and non-cavitated approximal caries lesions. Three studies have been carried out in primary teeth [12–14] and showed that both LFpen and bitewing radiography had a similar performance and capability to detect cavitation. A cut-off value of ≥17 demonstrated higher sensitivity and specificity in detecting approximal cavitation in primary teeth than a cut-off value of >16 (Appendix 1). However, such cut-off values for primary teeth cannot be extrapolated to permanent teeth, due to differences in the mineralisation degree between these two types of teeth [12–15]. Only one in vivo study evaluated the efficiency of the LFpen device in distinguishing between cavitated and non-cavitated approximal caries lesions in permanent teeth [15]. Nevertheless, this study has many limitations. Thus, the objectives of the current in vivo study were (1) to assess the diagnostic accuracy of the LFpen device in detecting approximal caries, in posterior permanent teeth, at the cavitation and non-cavitation thresholds, in a sample of individuals attending a dental hospital setting and (2) to compare the LFpen diagnostic accuracy with that of digital bitewing radiography.

## Subjects and methods

### Study design and sample selection

The current study adopted an observational prospective longitudinal design. With respect to sample size calculation, the only previous similar study [15] did not report the parameters that could be used to calculate the current study’s sample size i.e. the difference in the mean of LFpen readings between the three groups of approximal surfaces (intact, with white/brown spots and cavitated) and the standard deviation. Thus, it was not feasible to assess a priori these parameters for power calculation for the current study. To address this, the present study was preceded by a feasibility study (on 30 surfaces, 10 in each group) to estimate the aforementioned parameters. Based on the feasibility study, the sample size of 90 approximal surfaces of posterior permanent teeth, distributed equally in three groups (intact, with white/brown spots and cavitated), was estimated to detect statistically significant differences in the mean of LFpen readings of 5 or above (SD = 2.5) between the three groups. This calculation set the power of the test at 80% and the level of significance at 5%.

Thirty patients (aged 18–37 [mean = 20.9 (SD = 3.2)]), who attended the Faculty of Dentistry at Damascus University, for a dental examination before receiving treatment, were consecutively screened. Patients with severe systematic diseases or who received fluoride varnish care in the last 6 months were excluded. Exclusion criteria for surfaces were the presence of frank approximal cavitated caries lesions (e.g. the absence of a marginal ridge), blood, hypoplastic pits, restorations or crowns, large caries lesions on smooth or occlusal surfaces and the absence of an adjacent tooth.

Ethical approval for the current study protocol was obtained from the Damascus University Faculty of Dentistry Research Ethics Committee (no. 2703/2013). Written informed consent was obtained from all individual participants included in the study.

### Laser fluorescence examination

The selected approximal surfaces of premolars and permanent molars were assessed by the LFpen. They were cleaned with a slow rotating bristle brush and dental floss. A laser fluorescence device, DIAGNOdent pen (KaVo, Biberach, Germany), with an approximal tip (Probe tip 1) was used for laser fluorescence examination according to the manufacturer’s instructions. The LFpen device was first calibrated against the ceramic reference, the fluorescence of which is known. After the standard calibration, the fluorescence value of a sound spot was recorded (zero value) and subtracted later from the values assessed on the tooth surface [16]. After a standardised drying time of 5 s using compressed air, the tip of the LFpen device was placed underneath the contact area and then moved to the marginal ridge, first from the buccal side and then from the lingual/palatal side, recording the peak value [12, 14]. The measurement was repeated three times on the side that had a higher peak value [12]. The average of the three peak values from the same side was calculated. This value was recorded as the surface score that was subject to statistical analyses.

### Radiographic examination

For the radiographic examination, digital bitewing radiographs were made for posterior teeth using an intraoral sensor (Sopix USB, Acteon Group, La Ciotat, France) with a bitewing sensor holder (Kwik-Bite Senso, Kerr X-ray sensor holders, USA) and a paralleling long cone technique. The focus-to-sensor distance was about 40 cm. The intra-oral X-ray unit (X-Mind DC, Acteon Group, La Ciotat, France) was set at 70 kV, 8 mA, and the exposure time was 0.080 s.

A personal laptop (Fujitsu, LIFEBOOK AH 530) running Microsoft Windows 7 as an operating system with a 14-in. LCD screen in a dark room was used for interpreting the bitewing radiographs. The SOPRO Imaging software (Sopro, Acteon Group, La Ciotat, France) was used to view the radiographs. One digital radiograph with appropriate density and contrast was used as a standard reference for radiographic density and contrast against which all the current study radiographs were manipulated. The two radiographs (the reference and the radiograph under studying) were demonstrated together on the screen. The examiner adjusted the radiographic density (brightness) and the contrast of the radiograph under studying visually to get as much as the same density of the dental hard structures and the same contrast between the enamel and dentine of the reference radiograph. The scoring system suggested by Marthaler [17] was used. The frequency distribution of surfaces with radiolucency in the outer half of the enamel suggested collapsing original scores 1 and 2 (radiolucency in the outer and inner half of the enamel, respectively) into one group. Thus, the approximal surfaces were scored as follows: 0 = no radiolucency; 1 = radiolucency in the enamel; 2 = radiolucency in the outer half of dentine; and 3 = radiolucency in the inner half of dentine.

### Reference standard method

The reference (gold) standard method was performed 1 week after performing the LFpen device measurement and taking the digital bitewing radiograph, using the visual-tactile inspection. In order to perform the reference standard assessment, temporary separation was carried out for selected approximal surfaces using orthodontic rubber rings (American Orthodontics, USA), which were placed around the contact points for 7 days. In case of rubber loss or inadequate separation, the abovementioned separation procedure was repeated. After cleaning the separated surfaces, the surface was assessed with a dental mirror and a World Health Organization (WHO) periodontal probe (CPITN Probe) and classified as (i) sound (score 0 = no change in enamel translucency after air drying and absence of surface discontinuity), (ii) with white/brown spot lesion (score 1 = white or brown discoloration in wet or dried tooth with no enamel discontinuity) and (iii) with cavitated caries lesion (score 2 = loss of integrity of the surface detected visually and/or with the WHO probe) [13–15].

One trained examiner (RM), who is a dentist with 4 years of postgraduate training in dental radiology, carried out all the examinations. The examiner was trained to conduct the LF pen, radiographic and reference standard examinations by an experienced dentist and specialist in dental radiology (IA). The training included a 1-h theoretical session followed by two clinical sessions (3-h each). The clinical sessions involved examining nine surfaces (sound, with white/brown spots or cavitated). These surfaces were not included in the present study.

One week after all assessments, nine approximal surfaces (10% of the sample size, as conventionally recommended) were re-assessed, using the LFpen device, in order to calculate intra-examiner reliability for the LFpen readings. Also, nine digital bitewing radiographs were re-assessed to calculate the intra-examiner reliability for the radiographic assessment. Seven approximal surfaces were re-examined, using the visual-tactile inspection after temporary separation, in order to calculate intra-examiner reliability for the reference standard.

### Statistical analyses

Data were analysed using the Statistical Package for Social Science software (SPSS version 22, IBM Corp., Armonk, NY, USA) and MedCalc for Windows (version 14.8.1.0, Mariakerke, Belgium). Kruskal-Wallis and chi square tests were carried out to test the significance of the differences in the LFpen device’s readings and radiographic assessments, respectively, amongst the current study’s three groups of approximal surfaces: sound, with white/brown spots and cavitated surfaces. Thereafter, the sensitivity, specificity and accuracy values and the 95% confidence interval (95% CI) were calculated for the LFpen device and digital bitewing radiography at the cavitation and non-cavitation thresholds, using the receiver operating characteristic (ROC) analyses [18]. The cut-off values for the LFpen device were determined in a way that enabled the highest sum of sensitivity and specificity at the cavitation and non-cavitation thresholds [18]. The cut-off values for the digital bitewing radiography were determined based on the radiolucency in dentine and enamel at the cavitation and non-cavitation thresholds, respectively [19, 20]. The area under the ROC curve (AUC) was used to calculate the accuracy values of the two diagnostic methods and their *P* values [18]. A *P* value of <0.05 indicates the ability of the diagnostic method to distinguish between the cavitated and non-cavitated surface groups and between the intact and carious surface groups. The positive/negative predictive values were calculated [18]. The intra-examiner reliability for the LFpen readings was assessed using intra-class correlation coefficient (ICC; Spearman rank order correlation coefficient [rho]). The intra-examiner reliability for the radiographic assessment and visual-tactile inspection after temporary tooth separation was assessed using Cohen’s kappa coefficient. To compare the diagnostic performance of LFpen with that of digital bitewing radiography, the significance of the difference in the values of the AUC of both methods was tested using the normal approximation under the null hypothesis (i.e. the AUC of the LFpen = the AUC of the digital bitewing radiography). MedCalc software was used for this test. The level of significance was set at the 0.05 level for all statistical tests.

## Results

The response rate was 81.1%, and the dropout was 16.7%. Thirty patients (aged 18–37 [mean = 20.9 (SD = 3.2)]), of whom 41.9% were males, completed the current study. Eighteen patients had high risk of dental caries (the presence of at least one obvious [cavitated] current active caries [21]), whereas 12 patients had low risk of dental caries. Twenty-two patients participated in the reliability measurements.

The selected 90 approximal surfaces included 9 (10%) upper premolar, 18 (20%) lower premolar, 25 (27.8%) upper permanent molar and 38 (42.2%) lower permanent molar surfaces. These surfaces were distributed in three equal groups: 30 intact, 30 with white/brown spots and 30 cavitated surfaces. Two patients presented approximal surfaces with all conditions under study (i.e. intact, with white/brown spots and cavitated).

Table 1 summarises the LFpen device’s readings in the current study’s three groups. There was a significant difference in LFpen readings across the three approximal surfaces’ groups (*P* < 0.001) (Table 1). An inspection of the groups’ mean ranks suggested that the cavitated surfaces had the highest LFpen readings, with the intact surfaces reporting the lowest (Table 1).

**Table 1:** Laser fluorescence (LFpen) readings and the results of Kruskal-Wallis analysis to test the significance of the differences in LFpen readings amongst the current study’s three groups of approximal surfaces: sound, with white/brown spots and cavitated surfaces

Approximal surface’s status	Number	LFpen readings’ mean (SD)	Mean rank	*P* value
Intact	30	6.5 (2.5)	18.63	<0.001
With white/brown spots	30	15.1 (7.8)	45.20	
With cavitation	30	34.7 (16.1)	72.67	

**Fig. 1:**
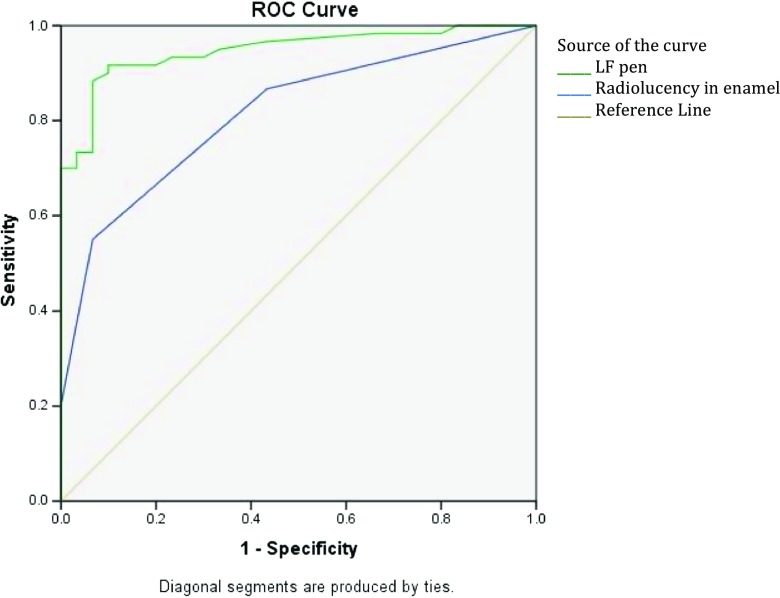
The receiver operating characteristic (ROC) curve of the diagnostic performance of the LFpen device (*green line*) and digital bitewing radiography (*blue line*) in detecting approximal cavitated caries lesions in posterior permanent teeth

At the cavitation threshold, Fig. 1 shows that the ROC curve followed very closely the left-hand border and then the top border of the ROC space, indicating high accuracy. The area under the curve indicated 0.95 (95% CI = 0.91–0.99; *P* < 0.001) accuracy of the LFpen device in detecting approximal cavitated caries lesions. The optimal cut-off value that enabled the highest sum of sensitivity and specificity at the cavitation threshold was >16. Based on this cut-off value, sensitivity and specificity were 100 and 85% respectively. The positive likelihood ratio rate was 6.7, and the negative likelihood ratio was 0, suggesting that this device is very/often useful for detecting approximal cavitated caries lesions. The ICC (rho = 0.95; 95% CI = 0.92–0.97) indicated excellent intra-examination reliability.

**Fig. 2:**
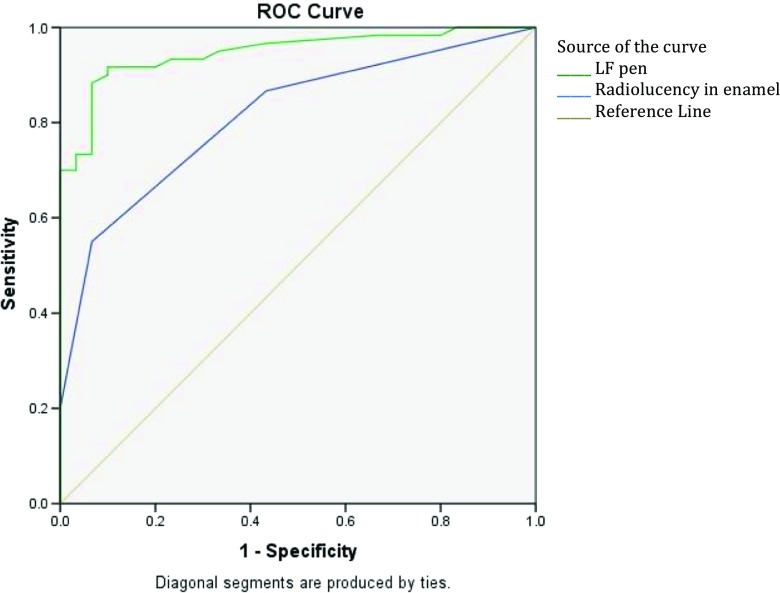
The receiver operating characteristic (ROC) curve of the diagnostic performance of the LFpen device (*green line*) and digital bitewing radiography (*blue line*) in detecting approximal caries lesions in posterior permanent teeth

At the non-cavitation threshold, Fig. 2 shows that the ROC curve followed also closely the left-hand border and then the top border of the ROC space, indicating high accuracy. The area under the curve indicated 0.95 (95% CI = 0.90–0.99; *P* < 0.001) accuracy of the LFpen device in detecting approximal caries lesions. The optimal cut-off value that enabled the highest sum of sensitivity and specificity at the non-cavitation threshold was 8. Based on this cut-off value, sensitivity and specificity were 92 and 90% respectively. The positive likelihood ratio rate was 9.2 and the negative likelihood ratio was 0.9, suggesting that this device is very useful for detecting approximal caries lesions.

With respect to the digital bitewing radiography, Table 2 summarises the frequencies of radiolucency scores in the present study’s three groups. There were significant differences in digital bitewing radiography readings across the three approximal surfaces’ groups (Table 2). The area under the curve indicated 0.78 (95% CI = 0.68–0.88; *P* < 0.001) and 0.81 (95% CI = 0.73–0.90; *P* < 0.001) accuracy of the digital bitewing radiography in detecting approximal caries lesions, at the cavitation and non-cavitation thresholds, respectively (Figs. 1 and 2). Sensitivity and specificity were 63 and %73 and 55 and 93%, at the cavitation and non-cavitation thresholds respectively. At the cavitation threshold, the positive likelihood ratio rate was 2.7 and the negative likelihood ratio was 0.5, suggesting that digital bitewing radiography is sometimes useful for detecting approximal cavitated caries lesions. At the non-cavitation threshold, the positive likelihood ratio rate was 7.9 and the negative likelihood ratio was 0.5, suggesting that digital bitewing radiography is often/sometimes useful for detecting approximal caries lesions. Cohen’s kappa coefficient was 0.81 (95% CI = 0.79–0.83), indicating excellent intra-examination reliability.

**Table 2:** Digital bitewing radiography frequencies and the results of chi-square analysis to test the significance of the differences in readings amongst the current study’s three groups of approximal surfaces: sound, with white/brown spots and cavitated surfaces

Approximal surface’s status	Score of bitewing radiography, *N* (%)				
	Total	0	1	2	3
Intact	30 (100)	17 (56.7)	11 (36.7)	2 (6.7)	0 (0)
With white/brown spots^a^	30 (100)	7 (23.3)	9 (30)	12 (40)	2 (6.7)
With cavitation^ab^	30 (100)	1 (3.3)	10 (33.3)	9 (30)	10 (33.3)

^a^Significantly different from the intact group (*P* ≤ 0.001)

^b^Significantly different from the white/brown spots group (*P* = 0.016)

Cohen’s kappa coefficient of visual inspection was 1 (95% CI = 1.00–1.00), indicating also excellent agreement.

Tables 3 and 4 summarise the comparison in the diagnostic accuracy between LFpen and digital bitewing radiography at the cavitation and non-cavitation thresholds respectively. The AUC was significantly higher in LFpen than in bitewing radiography with regard to detecting approximal caries lesions at both thresholds (*P* < 0.001).

**Table 3:** Comparing the diagnostic accuracy of the LFpen and digital bitewing radiography in detecting approximal cavitated carious lesion in posterior permanent teeth

Diagnostic method	Cut-off value	Sensitivity	Specificity	Accuracy	Reliability	*P* value^a^
LFpen	>16	100%	85%	95%	0.95	<0.001
Digital bitewing radiography	Radiolucency in dentine	63%	73%	68%	0.81	

^a^The difference in the values of the area under the (ROC) curve of both diagnostic methods

**Table 4:** Comparing the diagnostic accuracy of the LFpen and digital bitewing radiography in detecting approximal carious lesion in posterior permanent teeth

Diagnostic method	Cut-off value	Sensitivity	Specificity	Accuracy	Reliability	*P* value^a^
LFpen	8	92%	90%	95%	0.95	<0.001
Digital bitewing radiography	Radiolucency in enamel	55%	93%	81%	0.81	

^a^The difference in the values of the area under the (ROC) curve of both diagnostic methods

## Discussion

The present study demonstrated the diagnostic accuracy of the LFpen device in detecting approximal carious lesions, in posterior permanent teeth, in a sample of individuals attending a dental hospital setting. This diagnostic accuracy was significantly higher than that of digital bitewing radiography.

Previous in vivo studies that investigated the diagnostic accuracy of the LFpen device in discriminating between cavitated and non-cavitated approximal caries lesions were either performed on primary molars or included a sample of individuals with a low level of dental caries risk (Appendix 1). In both cases, findings cannot be extrapolated to permanent teeth or to the population of patients that attend dental care settings respectively. For example, Akbari et al.’s [15] study recruited dental undergraduates who are expected to have low risk of dental caries. This, in turn, implies that the identified cut-off value of ≥18 in this study cannot be generalised to the population that attends dental care settings and includes individuals with high and low levels of dental caries risk. In addition, Akbari et al.’s study included a very small sample size (only seven cavitated approximal caries lesions) and did not compare the performance of the LFpen with that of bitewing radiography. Thus, to the best of our knowledge, this is the first in vivo study that investigated the LFpen device’s diagnostic accuracy in detecting approximal caries lesions, in posterior permanent teeth, at the cavitation and non-cavitation thresholds, in a sample of individuals attending a dental care setting and compared the LFpen diagnostic accuracy with that of digital bitewing radiography against visual-tactile inspection as a gold standard.

Both our study and Akbari et al.’s [15] study showed a 100% sensitivity of the LFpen device in detecting approximal cavitated caries lesions in posterior permanent teeth, compared to a 55–92% sensitivity reported in studies conducted on primary molars [12–14] (Appendix 1). Also, the reliability reported in the current study and Akbari et al.’s [15] study was either similar or higher than that reported in studies conducted on primary molars (Appendix 1).

The optimal cut-off value for detecting approximal cavitated caries lesion in posterior permanent teeth was different than that identified by Akbari et al.’s [15] study. The latter selected the value of 18 as an optimal cut-off value, which is higher than the present study’s optimal cut-off value of >16. This difference might be due to the difference in dental caries risk levels of included subjects. Akbari et al. [15] recruited dental undergraduates who are expected to have a low level of dental caries risk, whereas our study recruited individuals attending a dental hospital setting, with high and low levels of dental caries risk. More likely, and as the difference is only 2 units, the latter might be due to the wide value variations the LFpen shows between individual measurements [22].

We recruited patients attending a dental hospital. Therefore, the cut-off value identified in the current study is more generalisable to the population of patients attending dental care settings. It is worth mentioning that this cut-off value is very close to the cut-off value identified by Huth et al. [23] and used by Kuhnisch et al. [24] in their in vivo studies to distinguish between enamel and dentine caries lesions in posterior permanent teeth.

There was a statistically significant difference in the LFpen readings amongst our study’s three types of approximal surfaces (intact, with white/brown spots and cavitated). The diagnostic accuracy of the LFpen device was also higher than that of digital bitewing radiology at the non-cavitation threshold too. Previous in vivo studies, in primary or permanent posterior teeth, have shown lower diagnostic performance of the LFpen device at the spot threshold than at the cavitation threshold [12–15]. Cavitated caries lesions are more infected than the non-cavitated ones [25, 26]; thus, a better performance of the LFpen device in detecting cavitated lesions is expected. The selection of the cavitation and non-cavitation thresholds in the present study is relevant to the preventive and operative care for patients.

The current study demonstrated that the performance of the LFpen device in detecting approximal cavitated caries lesions in posterior permanent teeth was significantly better (more accurate) than that of bitewing radiography. Previous in vivo studies that reported such comparisons in primary teeth showed similar performance of LFpen and radiographic methods [12–14]. Assessing the performance of the bitewing radiography method, in our study’s sample, showed lower sensitivity, specificity, accuracy, intra-examiner reliability and positive likelihood ratio and a higher negative likelihood ratio than that of the LFpen device. This might suggest that in permanent teeth, the diagnostic performance of the LFpen device can exceed that of bitewing radiography.

Previous in vivo studies did not attempt to measure the reliability of their gold standard. Whilst this is not feasible in Chen et al.’s [12] study, which used invasive treatment as a gold standard, the others, which used visual-tactile inspection after tooth separation as a gold standard, did not measure the reliability of this gold standard [13–15]. The present study found that visual-tactile inspection after tooth separation has the highest value of intra-examiner reliability compared to the LFpen and digital radiography.

One of the potential limitations of the current study is that only one trained examiner performed all examinations. In diagnostic accuracy studies, one potential source of bias is the availability of reference standard results to the performers/readers of the test method, as well as the availability of the test method results to the assessors of the reference standard [27]. Despite the present study’s efforts to minimise this potential bias by assessing the test methods and reference standard at different points of time and recording related data during assessment in separate examination forms, this potential bias cannot be completely ruled out.

The findings of this study could be considered as a first step in establishing the diagnostic accuracy of the LFpen device in detecting approximal caries lesions in posterior permanent teeth, at the cavitation and non-cavitation thresholds. Future epidemiological studies with larger sample size are needed to confirm the current findings that would have major implications in evidence-based dental practice guidelines. Also, future studies should include patient-centred outcomes, such as dental pain and discomfort and oral health-related quality of life. A previous study on primary teeth included a measure of pain and discomfort in children [14]. Including such outcomes is essential to draw appropriate conclusions and decisions.

In conclusion, the LFpen device is an accurate diagnostic method in detecting approximal carious lesions in posterior permanent teeth, at the cavitation and non-cavitation thresholds, in a sample of individuals attending a dental hospital setting. The diagnostic accuracy of the LFpen is significantly higher than that of digital bitewing radiography.

## Electronic supplementary material

Below is the link to the electronic supplementary material.

Appendix 1 Previous in vivo studies that investigated the diagnostic accuracy of the LFpen device and bitewing radiography in distinguishing between cavitated and non-cavitated approximal caries lesions (DOCX 19 kb)
