# Supplementary material for: The diagnostic accuracy of a laser fluorescence device and digital radiography in detecting approximal caries lesions in posterior permanent teeth: an in vivo study
Source: Lasers Med Sci. 2017 Feb 14;32(3):621–8. doi: 10.1007/s10103-017-2157-2 (PMC5360861; doi:10.1007/s10103-017-2157-2)
Supplement: Supplementary file 1 — Previous in vivo studies that investigated the diagnostic accuracy of the LFpen device and bitewing radiography in distinguishing between cavitated and non-cavitated approximal caries lesions (DOCX 19 kb) [file 10103_2017_2157_MOESM1_ESM.docx]

**Appendix 1:** Previous in vivo studies that investigated the diagnostic accuracy of the LFpen device and bitewing radiography in distinguishing between cavitated and non-cavitated approximal caries lesions.

| **Reference** | **Tooth type** | **LF pen** | | | | | **Bitewing Radiography** | | **Gold standard** |
| --- | --- | --- | --- | --- | --- | --- | --- | --- | --- |
|  |  | **Cut-off value** | **Sensitivity** | **Specificity** | **Accuracy** | **Reliability** | **Sensitivity** | **Specificity** |  |
| Novaes et al. (2009) | Primary molars | > 16 | 0.55-0.65 | 0.94-0.96 | 0.98-0.99 | 0.75 | 0.65-0.70 | 0.99 | VTI^a^ after TS^b^ |
| Novaes et al. (2010) | Primary molars | > 16 | 0.42-0.52 | 0.95 | 0.92-0.93 | 0.40-0.62 | 0.52 | 0.98 | VTI^a^ after TS^b^ |
| Chen et al. (2012) | Primary molars | ≥17 | 0.92 | 0.98 | 0.94 | 0.99 | 0.98 | 0.93 | Invasive treatment |
| Akbari et al. (2013) | Posterior permanent teeth | ≥18 | 1 | 0.97 | 0.98 | 0.96 | - | - | VTI^a^ after TS^b^ |

^a^ VTI = visual-tactile inspection.

^b^ TS = temporary separation.
